# STAT3-dependent long non-coding RNA Lncenc1 contributes to mouse ES cells pluripotency via stabilizing Klf4 mRNA

**DOI:** 10.1093/bfgp/elad045

**Published:** 2023-10-04

**Authors:** Emanuele Monteleone, Paola Corrieri, Paolo Provero, Daniele Viavattene, Lorenzo Pulvirenti, Laura Raggi, Elena Carbognin, Marco E Bianchi, Graziano Martello, Salvatore Oliviero, Pier Paolo Pandolfi, Valeria Poli

**Affiliations:** Department of Molecular Biotechnology and Health Science, University of Torino, Via Nizza 52, 10126 Torino, Italy; Università Vita-Salute San Raffaele, Milan, Italy; Department of Molecular Biotechnology and Health Science, University of Torino, Via Nizza 52, 10126 Torino, Italy; Department of Molecular Biotechnology and Health Science, University of Torino, Via Nizza 52, 10126 Torino, Italy; Department of Molecular Biotechnology and Health Science, University of Torino, Via Nizza 52, 10126 Torino, Italy; Department of Molecular Biotechnology and Health Science, University of Torino, Via Nizza 52, 10126 Torino, Italy; Department of Molecular Biotechnology and Health Science, University of Torino, Via Nizza 52, 10126 Torino, Italy; San Raffaele-Telethon Institute for Gene Therapy (SR-TIGET), Milan, Italy; Department of Biology, University of Padua, Italy; Università Vita-Salute San Raffaele, Milan, Italy; Department of Biology, University of Padua, Italy; Department of Life Sciences and Systems Biology, University of Torino; Department of Molecular Biotechnology and Health Science, University of Torino, Via Nizza 52, 10126 Torino, Italy; William N. Pennington Cancer Institute, Nevada System of Higher Education, Reno, Nevada; Department of Molecular Biotechnology and Health Science, University of Torino, Via Nizza 52, 10126 Torino, Italy

**Keywords:** STAT3, lincRNAs, Klf4, microRNAs, miR-128, naive pluripotency

## Abstract

Embryonic stem cells (ESCs) preserve the unique ability to differentiate into any somatic cell lineage while maintaining their self-renewal potential, relying on a complex interplay of extracellular signals regulating the expression/activity of pluripotency transcription factors and their targets. Leukemia inhibitory factor (LIF)-activated STAT3 drives ESCs’ stemness by a number of mechanisms, including the transcriptional induction of pluripotency factors such as Klf4 and the maintenance of a stem-like epigenetic landscape. However, it is unknown if STAT3 directly controls stem-cell specific non-coding RNAs, crucial to balance pluripotency and differentiation. Applying a bioinformatic pipeline, here we identify Lncenc1 in mouse ESCs as an STAT3-dependent long non-coding RNA that supports pluripotency. Lncenc1 acts in the cytoplasm as a positive feedback regulator of the LIF–STAT3 axis by competing for the binding of microRNA-128 to the 3’UTR of the Klf4 core pluripotency factor mRNA, enhancing its expression. Our results unveil a novel non-coding RNA-based mechanism for LIF–STAT3-mediated pluripotency.

## INTRODUCTION

Mouse embryonic stem cells (ESCs) are pluripotent cells established from the early epiblast of pre-implantation embryos, able to differentiate into any of the three germ layers. ESCs are endowed with *in vitro* self-renewal features generating, through symmetric cell divisions, pluripotent daughter cells closely related to the pluripotent naive state of the inner cell mass of blastocysts [1]. ESCs stemness is maintained by a regulatory circuitry involving several so-called pluripotency transcription factors, i.e. Oct3/4, Sox2, Klf4, Nanog and Myc. Not only these act in a coordinated way to activate specific gene programs central to the establishment and maintenance of ESC identity, but they are also able to force direct reprogramming of somatic cells into induced pluripotent stem cells (iPSCs) [2]. Additionally, a number of non-coding RNAs have been shown to contribute to the regulation of ESCs pluripotency, including microRNAs and long non-coding (lnc) RNAs [3, 4]. LncRNAs have indeed emerged as an important class of gene expression regulators, often displaying strict cell- and tissue-specific patterns [5], suggesting their participation in the intricate transcriptional/post-transcriptional ESC network [6].

The maintenance of ESCs self-renewal involves a complex balance between pro- and anti-differentiation mechanisms, triggered by extracellular signals. In particular, self-renewal of murine ES cell lines requires leukemia inhibitory factor (LIF), which, in turn, activates the JAK–STAT3 pathway [7]. STAT3 is a pleiotropic transcription factor activated by many different cytokines, growth factors and oncogenes, playing multifaceted roles in regulating cell proliferation, apoptosis, migration and differentiation [8]. Being often constitutively activated in tumors of both solid and liquid origin, STAT3 is considered as an oncogene, and indeed its forced activation is able to induce tumor transformation [9, 10]. Several data confirm the crucial role played by STAT3 in murine ESCs self-renewal. STAT3 inactivation leads to ESCs spontaneous differentiation [11, 12] and, conversely, its constitutive expression is sufficient to maintain ESCs pluripotency, bypassing the need for LIF in the culture medium [13]. Different from mESCs, which maintain a naive state in culture, human ESCs are in a primed state, expressing lower levels of the core pluripotency factors and several epiblast markers [14]. Accordingly, hESCs do not require LIF but Fibroblast Growth Factor-2 and activin/nodal signaling for self-renewal maintenance. However, transient reinforcement of STAT3 activity can reprogram hESCs to a naive state more closely resembling that observed in their murine counterpart [15], showing that the LIF–STAT3 axis is an important regulator of pluripotency in both human and mouse ESCs.

STAT3 acts at different levels to maintain self-renewal *via* the activation of multiple targets. First, it takes part in the transcriptional network formed by the key pluripotency factors, some of which (c-Myc and Klf4) are STAT3 transcriptional targets. STAT3 is also known to regulate chromatin structure via metabolic control of DNA methylation [16], and to mediate a cross-talk with other pathways such as, for example, the Wnt signaling, which acts synergistically with LIF via their converging effects on β-catenin [17].

Deeper insights in the activities of STAT3 in ESCs may help further dissecting the molecular mechanisms regulating self-renewal and possibly unveiling novel molecular targets mediating STAT3-dependent pro-oncogenic activities, first and foremost the induction of a stem cell status in cancer cells [18]. Despite abundant data describing STAT3-mediated regulation of protein coding genes, its association with regulatory non-coding RNAs, in particular long non-coding RNAs, in ESCs are poorly studied. Here, we identify a subset of putative STAT3-dependent ESC-specific long intergenic non-coding (linc) RNAs and characterize the role of one of them, Lncenc1, revealing that its silencing triggers mESCs differentiation, at least partly by outcompeting the 3’UTR of Klf4 for the binding of microRNA-128.

## MATERIALS AND METHODS

### Cell culture and differentiation

E14 mouse ESCs were cultured without feeders on plastic coated with 0.1% gelatin (Sigma, cat. G1890) and reseeded every 2/3 days at a split ratio of 1 in 10. Cells were cultured either in high-glucose DMEM (Thermo Fisher Scientific, Waltham, MA, USA) supplemented with 15% FBS (Hyclone, Logan, UT, USA), 0.1 mM non-essential amino acids (ThermoFisher Scientific), 1 mM sodium pyruvate (ThermoFisher Scientific), 0.1 mM 2-mercaptoethanol, 1500 U/ml LIF (Merck Millipore, Darmstadt, Europe), 25 U/ml penicillin and 25 μg/ml streptomycin, or in the serum-free medium N2B27 (Invitrogen) supplemented with the small-molecule inhibitors PD (1 μM, PD0325901) and CH (3 μM, CHIR99021) and LIF. Embryoid bodies (EBs) were generated as follows. E14 cells were dissociated in trypsin and resuspended at a concentration of 3 × 10^4^ cells/ml in ES cell medium without LIF. 30 μl hanging drops containing 900 mES cells were incubated for 3 days. EBs were harvested in 60 mm low attachment plates in ES cell medium without LIF, and incubated for an additional 3 days. EpiSCs GOF18 were cultured as previously described [19].

E14 cells stably overexpressing Klf4 were generated by co-transfecting the plasmid phyPBase, expressing a PiggyBac hyperactive transposase [20], and the Klf4 expression vector pGG137_mKlf4 [21]. 24 hours post transfection, cells were selected for 6 days with 200 μg/ml of Hygromycin B (Sigma).

### Alkaline phosphatase assay

ES cells colonies were fixed with 4% paraformaldehyde (PFA), followed by two PBS washes and an O/N incubation in PBS. Upon taking phase contrast pictures, AP staining was performed using the Vector® Red Substrate Kit (Vector Laboratories, Newark, USA) according to manufacturer’s instructions. To score AP staining and colonies circularity, imaged cells were analyzed using a custom macro in the FIJI software (https://imagej.net/software/fiji).

### Plasmid constructs

Custom shRNAs were designed using the TRC hairpin design tool (http://www.broadinstitute.org/rnai/public/seq/search). shRNAs with more than four consecutive matches to non-target transcripts were avoided. Hairpins were cloned into the pLKO.1 vector (Addgene, Watertown, MA, USA: Cat. No. 10878) and controlled by sequencing. The pLKO.1 non-targeting control vector was purchased from Addgene (Cat. No. 136035). The MREs enriched region of Lncenc1 was amplified by PCR from E14 genomic DNA purified using QIAquick Gel Extraction Kit_(Qiagen, Hilden, Germany, Cat. No. 28706X4) and cloned in the pLVX-Tight-Puro vector. The pPyCAGSTAT3ERT2iresZeo construct (STAT3ERT) was kindly provided by Austin Smith [7]. For luciferase constructs, the Klf4 3’UTR was amplified by PCR from E14 genomic DNA and cloned in the pMIR-REPORT vector (Thermo Fisher Scientific, Cat. No. AM5785). Site-directed mutagenesis on the resulting plasmid was performed using the Quick-Change kit (Stratagene, La Jolla, CA, USA, Cat. No. #200523). All primers used for cloning and mutagenesis are listed in Supplementary Table 1.

### Transfection

Transfection of E14 cells was performed using the LipofectamineTM 2000 Transfection Reagent (Thermo Fisher Scientific), using 10 μl of transfection reagent, 4 μg of plasmid DNA and 800 000 cells in 800 μl of OPTIMEM (Thermo Fisher Scientific) for each well of a six-well plate. After 6 hours, cells were rinsed with 1.2 ml of complete ESCs medium, incubated O/N, detached and seeded into a 10 cm plate. Unless otherwise noted, shRNA transfected cells were selected with 1 μg/ml of puromycin for 48 hours, followed by cell lysis at 72 hours, whereas ASOs-treated cells were analyzed 36 hours after removal of the transfection medium.

### RNA extraction and real time PCR analysis

Total RNA was extracted by using TRIzol reagent (Thermo Fisher Scientific), according to manufacturer’s protocol, cDNA generated using the High-Capacity cDNA Reverse Transcription Kit (Applied Biosystems, Waltham, MA, USA, Cat. No. #4375575), and Real-time PCR performed using the Fast SYBR™ Green Master Mix (Thermo Fisher Scientific), with the primers listed in Supplementary Table 5. For miRNAs quantification, cDNAs were generated using the Taqman Advanced miRNA cDNA Synthesis Kit (Applied Biosystems, Cat. No. A28007), and Real-time PCR performed on total RNA with the indicated TaqMan MicroRNA Assay (Applied Biosystems), according to manufacturer’s instructions, and normalized on U6 RNA levels.

### Lentivirus production and titration

Lentivirus production was performed using the Lenti-XTM Lentiviral Expression Systems (Clontech Laboratories, Mountain View, CA, USA). LentiX 293 T-Rex cell lines were transfected using Effectene Transfection Reagent (Qiagen) according to the manufacturer’s protocol. Lentiviral particles were harvested at 24 and 48 hours, filtered through a 0.22-μm pore cellulose acetate filters, concentrated by ultracentrifugation for 2 hours at 22,000 g and resuspended in 1× PBS, 1% BSA. Vector infectivity was evaluated by transducing ES cells with serial 4-fold dilutions: undiluted, 1/4, 1/16, 1/64, 1/256 and 1/1024. After 72 h, the titer was estimated by real-time quantitative RT-PCR of a common lentiviral genome region (WPRE), upon collection of the SN and RNA extraction.

### Chromatin immunoprecipitation assay

Chromatin immunoprecipitation was performed as described in Avalle *et al*. (2022) [22], with modifications. Briefly, 2^*^10^7^ cells were cross-linked with 1% formaldehyde for 10 minutes at RT, quenched with 0.125 M glycine for 5 minutes, and then washed twice in cold PBS. The cells were suspended in Lysis Buffer 1 (50 mM Hepes-KOH pH 7.5, 140 mM NaCl, 1 mM EDTA, 10% Glycerol, 0.5% NP-40, 0.25% Triton X-100 and protease inhibitor) to disrupt the cell membrane, followed by Lysis Buffer 2 (10 mM Tris–HCl pH 8.0, 200 mM NaCl, 1 mM EDTA, 0.5 mM EGTA and protease inhibitor) to isolate nuclei. The isolated nuclei were then resuspended in SDS ChIP Buffer (20 mM Tris–HCl pH 8.0, 10 mM EDTA, 1% SDS and protease inhibitors). Extracts were sonicated using the Bioruptor H Twin (Diagenode, Liege, Belgium) for two runs of 10 cycles [30 sec ‘ON’, 30 sec ‘OFF’] at high power setting. Nuclear lysates were centrifuged at 12,000 g for 10 minutes at 4°C. The supernatant was diluted with ChIP Dilution Buffer (20 mM Tris–HCl pH 8.0, 150 mM NaCl, 2 mM EDTA, 1% Triton) before the immunoprecipitation step. Streptavidin beads (Dynabeads®Protein G, Thermo Fisher Scientific) were saturated with PBS/1% BSA and samples were incubated with 5 μg of antibody overnight at 4 °C on a rotator. The antibodies used were anti-STAT3 antibodies (Cell Signaling Technology, Danvers, USA, Cat. No. 9132), phospho (S5) RNA polymerase II, ab5408 (Abcam, Cambridge, UK) or rabbit IgG (Thermo Fisher Scientific, Cat. No. 31235). Samples were then incubated with saturated beads for 2 hours at 4 °C on a rotator. Immunoprecipitated complexes were washed five times with RIPA buffer (50 mM Hepes-KOH pH 7.6, 500 mM LiCl, 1 mM EDTA, 1% NP-40, 0,7% Na-Deoxycholate) at 4 °C for 5 minutes each on a rotator. Elution Buffer was added and incubated at 65°C for 15 minutes. The de-crosslinking was performed at 65 °C overnight, followed by standard phenol:chloroform:isomyl alcohol (25:24:1) DNA purification and real time PCR (see ‘RNA extraction and Real Time PCR analysis’). Primers for SYBR green qPCR reactions are listed in Supplementary Table 5.

### RNA fluorescence *in situ* hybridization

A single long RNA biotinylated probe targeting Lncenc1 RNA was obtained as follows: a region of 542 bp was PCR amplified from genomic DNA with primers listed in Supplementary Table 5. The resulting DNA fragment was cloned into the pGEM T-easy vector (Promega Corporation, Madison USA, Cat. No. A1360). The resulting construct was used as a template for T7-based *in vitro* transcription using the MEGAscript™ T7 Transcription Kit (Thermo Fisher Scientific, Cat. No. AMB13345). *In situ* hybridization was performed as follows. Briefly, mESCs were seeded (3x10^5^) onto poly-L-lysine and gelatin coated glass coverslips, rinsed with PBS and fixed in 4% PFA. Upon incubating in 2× SSC, 1% BSA saturation buffer for 3 hours at room temperature, 10 ng of biotin-labeled probes diluted in hybridization buffer (2× SSC, 10% Deionized Formamide, 50% Dextran Sulfate) were heat denatured and incubated with the cells at 37°C O/N in a humidified chamber. All buffers used prior to hybridization were supplemented with RNAse inhibitors (SUPERaseIn, Thermo Fisher Scientific, Cat. No. A2694) at a concentration of 100 U/ml. Cells were then rinsed twice in either Wash buffer (2% SSC,50% Deionized Formamide) or PBS. Primary antibodies conjugated or not with streptavidin-PE (BioLegend, San Diego, CA, USA) were diluted 1:200 in blocking buffer (PBS 1X, 1% BSA). Biotinylated Anti-Streptavidin antibodies (Vector Laboratories, Cat. no. #BA-0500, 2 μg) were incubated for 1 hour at 37 °C, followed by three washes and 1 hour incubation with AlexaFluor-488-conjugated secondary antibodies (Thermo Fisher Scientific), diluted 1:200 in the Blocking buffer. DAPI (0.5 mg/ml) was used to visualize cell nuclei. Digital images were captured on a Leica TCS SP5 confocal system (Leica Microsystems, Wetzlar, Germany).

### Protein extraction and western blot

Total protein extracts were obtained and Western blots performed as previously described [23]. Antibodies were purchased either from Cell signaling (Tyr705 Phospho STAT3, #9145, STAT3 #9132), Abcam, Cambridge, UK (KLF4, ab72543, DICER1, ab227518), or Santa Cruz Biotechnology, Dallas, USA (Actin, Hsp90). Western blot chemiluminescence signals were acquired with a ChemidocTouch and analyzed with the ImageLab software (BioRad). All blots were repeated at least three times, and representative images shown.

### ChIP-seq data analysis

STAT3 and GFP control ChIP-seq data were from a previous study [24]. Reads were mapped to the mouse genome (build mm10) using bowtie 1 [25], discarding non-unique alignments. Peak detection was performed using MACS version 1.4.1 [26]. Each peak was associated with up to two genes as follows: the most proximal gene on each strand was identified by examining 5 kb upstream and downstream of the peak. For all other transcription factors, peaks were associated with the closest protein-coding by examining a window of 1 kb upstream the transcription start site. Overlaps between gene lists were evaluated using Fisher exact test and plotted using the ggplot2 R package (https://ggplot2.tidyverse.org/).

### RNA-sequencing

mES-E14 cells were lysed in TRIzol (Thermo Fisher Scientific). Total RNA integrity was assayed using a Bioanalyzer 2100 (Agilent, Santa Clara, CA, USA). All samples had RIN > 9. RNA-seq library preparation was performed starting from 2μg of total RNA and used as input for First Strand synthesis, using the TruSeq RNA Library Prep kit, following manufacturer instructions. Next-generation RNA sequencing was performed on Illumina HiScanSQ Platform. Reads overlapping annotations were counted with HTSeq-count suite (http://www-huber.embl.de/HTSeq) and Differential expression (DE) analysis was performed using the R package DESeq2 (https://bioconductor.org/packages/release/bioc/html/DESeq2.html). Genes with an adjusted *P*-value (false discovery rate, FDR) of <0.05 and with a log2 fold change major of the absolute value of 1 were considered as differentially expressed. RNA-Seq time-course analysis was performed using the Likelihood ratio test (LRT) and *P*-values were corrected using the Benjamini–Hochberg method.

Gene Ontology was performed using the topGO R package (https://bioconductor.org/packages/release/bioc/html/topGO.html). Heatmap and Hierarchical clustering was performed using the Heatmap.2 function of the ‘gplots’ R package. Sequencing Data are available on the gene omnibus (GEO) database under the accession ID number: GSE221670.

Public RNA-Seq Bam files were downloaded from ENCODE (https://www.encodeproject.org) and gene expression was quantified using Cufflinks v2.0.2 (http://cole-trapnell-lab.github.io/cufflinks/releases/v2.0.2/). To create a comprehensive and combined annotation set, we used the GFFcompare utility (https://ccb.jhu.edu/software/stringtie/gffcompare.shtml) on RefSeq, UCSC and Gencode annotation sets. LincRNAs displaying an FPKM below 1 were excluded from further analysis.

Tissue specificity score (SPM) was calculated as described in Xiao *et al.* (2010) [27]. ES-specific lincRNAs were obtained by performing Kmeans clustering analysis on SPM scores.

### miRNA target prediction

For miRNA target prediction we used the TargetScan software, release 6 (https://www.targetscan.org/mmu_60/). Annotated Klf4 3’UTR and Lncenc1 RNA was scanned for strict miRNA targets (8mer, 7mer-m8, and 7mer-A1). Evolutionary conserved mouse miR-128 and miR-138 binding sites in genes expressed in ES-cell were downloaded from the TargetScan database (release 6) and the probability of getting a number of miR128/138 binding sites was calculated using the Poisson distribution (*λ* is the mean number of miR128/138 binding sites per kilobases in the ES transcriptome).

### Statistical analysis

Unless otherwise noted, data were analyzed by one-way ANOVA or Students t-test, using the Prism (GraphPad software, Inc., San Diego, CA, USA). For RT-qPCR, statistical analysis were performed in 2-ΔΔCT values.

### Luciferase assays

Luciferase assays were all performed using the Dual-Luciferase® Reporter Assay System (Promega Corporation) and samples were acquired with the GloMax® plate reader. Data were normalized on the Renilla luciferase internal control.

## RESULTS

### Identification of a subset of putative STAT3-dependent ESC-specific long intergenic non-coding RNAs

**Figure 1:**
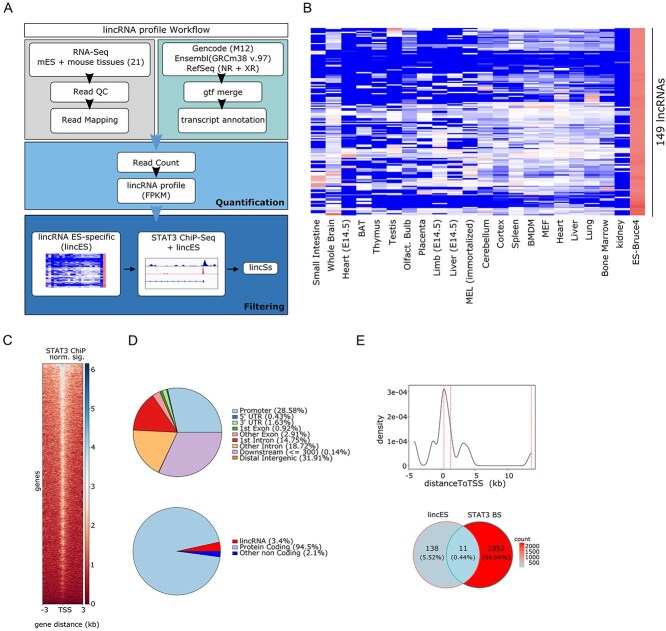
Identification of putative STAT3-dependent ESC-associated long intergenic non-coding RNAs (lincS). (**A**) Bioinformatics pipeline for ESC and mouse tissues transcriptome analysis. (**B**) Heatmap (GeneWise Z-score) representing the expression levels of the 149 lncRNAs identified as most specifically expressed in mESCs, in the indicated tissues or in Bruce ESCs (ENCODE RNA-sequencing data). (**C**) Heatmap showing the normalized STAT3-binding signal around the TSS (−3 to 3 kb) of genes expressed in mESC. (**D**) Genomic distribution of STAT3. Top panel: percentage of STAT3 binding at the indicated genomic locations. Bottom panel: STAT3 locations at promoters (~28.6% of all peaks) was further dissected according to the corresponding gene biotype (coding or non-coding). (**E**) Top panel: density distribution of the distance of STAT3-binding sites to the TSS of associated genes (see materials and methods for the peak–gene association rule). The distance between three STAT3-binding peaks and the TSS of lincS3 lincRNA is indicated by dotted lines intersecting the curve. Bottom panel: overlap between STAT3 peaks and putative regulatory regions of ESC-associated lincRNAs, identifying 11 lincSs.

To identify novel STAT3-dependent lincRNAs potentially implicated in self-renewal and/or pluripotency, we firstly selected those expressed in strong association with the stem cell state. To this end, we analyzed the raw poly(A) + RNA-seq data from the Bruce4 and E14 mouse ES cell lines along with 21 different mouse tissues released by the ENCODE Project (Figure 1A). Prior to mapping, we derived a comprehensive and up-to-date annotation set by merging gene features from GENCODE, Ensembl and RefSeq genesets. Following identical processing, we created a Fragments Per Kilobase of Exon per Million Fragments Mapped (FPKM)-based expression matrix, and for each gene, specificity scores were calculated (see materials and methods).

We then selected lincRNAs exclusively expressed in ESCs (lincES), with the exception of a few that were also detected in a single tissue, considered as potential biomarkers of development (Figure 1B). To identify putative STAT3-regulated lincRNAs, we re-analyzed publicly available STAT3 ChIP-Seq data in mouse ES cells [24]. The analysis of STAT3 genomic occupancy at both coding and non-coding genes expressed in ESC revealed a high preference for promoters (~28.6%, Figure 1C, D), distal intergenic (~32%) and intronic regions (first intron ~15%, ~19% other intron), while only 6% were within exons, both coding and non-coding (Figure 1D, top panel). Among promoter STAT3 binding sites, ~95% were linked to protein-coding genes, while ~5% were assigned to lincRNAs (~3.5%) or other non-coding genes (2%) (Figure 1D, bottom panel). To obtain a list of putative STAT3-dependent lincRNAs, we included lincRNA transcriptional units nearest to a detected STAT3 binding site, within +/−5 kb from the peak summit. Interestingly, the majority of peaks mapped relatively near the corresponding TSS, with only lincS3 displaying three STAT3 binding sites, at both proximal and distal sites (Figure 1E, top panel). We identified 11 lincRNAs displaying *in vivo* STAT3 binding at their putative regulatory regions, which we named STAT3-dependent lincRNAs (lincS) (Supplementary Table 1 and Figure 1E, bottom panel).

We focused our attention on LincS3, on the ground of its high expression levels and highest enrichment of STAT3 *in vivo* binding. This lincRNA (Lncenc1) was previously identified as a putative regulator of stem cell functions in several high throughput RNAi screenings [3, 28–30]. However, nothing is known about its functions along the LIF–STAT3 axis.

**Figure 2:**
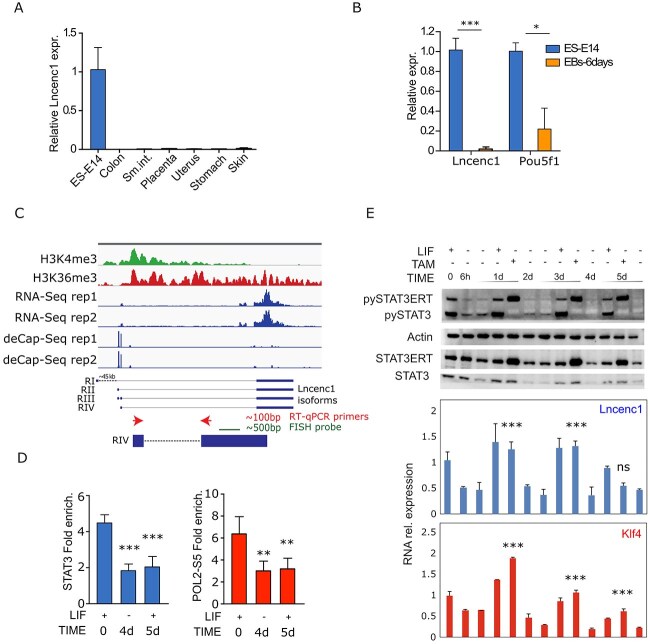
Lncenc1 is a STAT3-dependent ESC-specific lncRNA. (**A**, **B**) Lncenc1 and/or Pou5f1 (Oct4) RNA levels were measured by RT-qPCR in E14 mESCs and (A) the indicated adult mouse tissues, or (B) in derived EBs. Data are represented as the mean ± SD of the values normalized to the 18S rRNA internal control. *N* = 3. The asterisks indicate statistically significant differences. ^*^^*^^*^P < 0.0001; ^*^P < 0.01. (**C**) Integrative Genomics Viewer genome browser picture at the Lncenc1 locus showing the distribution of the H3K4 and H3K36 me3 chromatin marks, RNA-Seq profiles and deCAP-Seq data in E14 ES cells. Below, the RefSeq Lncenc1 isoforms RI–RIII are shown, together with the novel RIV isoform predicted by the deCap experiments. The RT-qPCR primers (arrows) and the probe used in the RNA FISH and Northern blot experiments are also shown. (**D**) ChIP-qPCR analysis showing STAT3 or PolII-PS5 relative enrichment on the Lincenc1 regulatory region and promoter, respectively, performed in E14 ESCs before or after LIF withdrawal (4 days) and upon 24 hours LIF re-supplementation (5 days). Data are mean ± SD of three independent experiments. ^*^^*^^*^P < 0.001; ^*^^*^P < 0.01. (**E**) STAT3-ERT E14 ESCs were deprived of LIF for 6 hours, 2 or 4 days, followed by 24-hour LIF or Tamoxifen re-stimulation prior to protein and RNA extraction. Top panel: Western blot obtained with either total or pYSTAT3 antibodies at the indicated time points. Actin was used as an internal control. The bottom panels show Lincenc1 and Klf4 RNA expression levels measured by RT-qPCR in the same samples, as the mean ± SD of three independent experiments. The asterisks indicate statistically significant difference between LIF-deprived samples, either treated or not treated with TAM at the indicated time points; ^*^^*^^*^P < 0.001; ns, not significant.

### The ESC-specific LincS3/Lncenc1 RNA is a STAT3 target

Lncenc1 ESC-specific expression was confirmed by RT-qPCR analysis, confirming that this RNA is undetectable in adult tissues or in EBs but highly expressed in mESCs (Figure 2A, B). We next sought to characterize the Lncenc1 locus, of which Figure 2C shows the structure and epigenetic status in ESCs. Three different Lncenc1 isoforms are reported in the RefSeq database, RI, RII and RIII, all sharing the last and largest exon that indeed shows the highest RNA-Seq coverage. However, analysis of mESCs deCAP-Seq [31], RNA-Seq, H3K4me3 and H3K36me3 histones methylation ChIP-Seq data (The ENCODE Project Consortium 2012) revealed a fourth prevalent isoform, RIV, with an alternative transcription start site but sharing the common largest exon. Targeted RT-PCR in E14 mESCs failed to detect the RI isoform and confirmed the prevalence of the RIV isoform (Supplementary Figure 1A). Accordingly, Northern blot analysis detected a single band, compatible with the predicted 3.3 kb molecular weight of the RIV isoform (Supplementary Figure 1B). The RT-PCR primers and probe used in the following experiments were therefore designed on this isoform (Figure 2C, bottom).

Two of the three STAT3 binding sites detected at the Lncenc1 locus (Figure 1E) mapped near the first exon of the RI isoform, while the third one was within the largest exon. Given that we could not detect any expression of the RI transcript and that the proximal STAT3 peaks are ~45 kb distal with respect to the RIV isoform, we focused on this latter binding site, where we could confirm LIF-dependent STAT3 *in vivo* binding by Chromatin Immunoprecipitation (ChIP) (Figure 2D). Further confirming STAT3-mediated transcriptional regulation, also active Serine 5 phosphorylated RNA Polymerase II binding was detected at the Lncenc1 promoter region, and was impaired upon LIF withdrawal (Figure 2D). Of note, the Lncenc1 locus is embedded in a previously reported super-enhancer region in mESCs [32], suggesting that STAT3 might orchestrate DNA looping to regulate Lncenc1 transcription.

To further assess transcriptional dependency on STAT3, we took advantage of previously generated E14 mESCs stably expressing a tamoxifene-dependent STAT3-ERT fusion protein [7]. Total RNA and protein extracts were obtained from these cells upon LIF deprivation for 6 hours, 2, 3 or 4 days, followed by supplementation with either LIF or Tamoxifene for 24 hours. As expected, tyrosine phosphorylation of both endogenous and recombinant STAT3 dramatically dropped after 6 hours of LIF withdrawal, became undetectable after 4 days and increased again upon either LIF or tamoxifen treatment, respectively (Figure 2E, top panel). Lncenc1 RNA levels closely paralleled STAT3-YP, being strongly decreased already six hours after LIF withdrawal and induced again by either LIF or Tamoxifen treatment up to 3 days after LIF deprivation (Figure 2E, bottom panel). At day 4, however, despite equivalent STAT3 phosphorylation levels, Lncenc1 expression could be no longer rescued, suggesting that differentiation had reached a point of no return. Similarly, neither LIF nor Tamoxifene re-supplementation could rescue STAT3 and PolII binding, suggesting an epigenetic mechanism impeding STAT3 binding and transcription (Figure 2D). Remarkably, the mRNA for the core pluripotency factor Klf4, a well-known LIF–STAT3 direct transcriptional target, closely paralleled Lncenc1 expression patterns (Figure 2E, bottom panel). Thus, both Lncenc1 and Klf4 mRNA levels similarly respond to STAT3, independently of LIF.

### Lncenc1 is associated with naïve pluripotency

**Figure 3:**
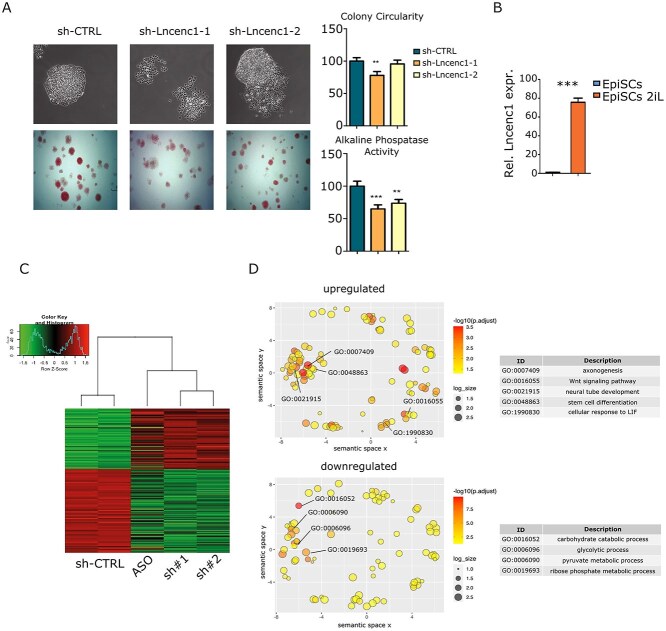
Lncenc1 expression correlates with stemness/naïve ES cells features. (**A**) Lncenc1 was silenced in E14 cells with two independent shRNAs (sh-Lncenc1-1 and -2) or with a scrambled control (sh-CTRL), followed by morphology analysis in phase contrast images (top left panel) and quantification of colony circularity as a measure of morphology disruption (top right panel). Alkaline phosphatase activity was measured and quantified (bottom panels). Data are mean ± SEM of four independent replicates. The asterisks indicate statistically significant difference between the indicated groups; ^*^^*^P < 0.001; ^*^^*^^*^P < 0.0001. (**B**) Relative expression of Lncenc1 RNA in mouse epiblast stem cells (EpiSCs) grown in 2i or reprogrammed toward the naïve state by culturing in 2i + LIF (2iL). ^*^P < 0.01; ^*^^*^P < 0.001; ^*^^*^^*^P < 0.0001. (**C**) Heatmap showing hierarchical clustering of differentially expressed genes (FDR < 0.05) in E14 cells following Lincenc1 knockdown by means of ASO, shRNA-1 or -2, or negative controls. (**D**) The REViGO Scatterplot shows the Enriched GO Clusters representative of genes up- or down-regulated in all three knock-down samples. GO terms along with their *P*-values are summarized in a two-dimensional space. Correlated GO terms cluster together. Bubble color indicates the *P*-value, as shown, and size the number of genes falling in that particular GO term. The definition of the selected GO terms indicated is shown in the corresponding tables.

To dissect its biological role, we depleted Lncenc1 by transfecting E14 ESCs with two independent shRNAs or a non-targeting control (Supplementary Figure 2A). Lncenc1 silencing triggered ESCs differentiation, as shown by disrupted colony morphology, quantified as colony circularity, and the significant reduction of Alkaline Phosphatase (AP) activity, a well-recognized pluripotency marker (Figure 3A). Further supporting its role in the maintenance/induction of pluripotency, Lncenc1 levels were dramatically induced upon reprogramming of epiblast stem cells toward a naive, ground state via culturing in 2i medium plus LIF (Figure 3B).

We then compared the RNA expression profiles of E14 ESCs silenced or not for Lncenc1 for 72 or 36 hours, respectively, by means of either shRNAs or Antisense Oligonucleotides (ASO) (Supplementary Figure 2B). Since ASOs are known to elicit quicker but more transient silencing as compared to shRNAs (Watts and Corey 2012, and data not shown), we reasoned that this strategy would allow us to assess both early and late silencing effects. Comparing two separate time points and two distinct silencing methods may in fact allow to better discern direct phenotypic effects. Bioinformatic analysis of RNAs dysregulated by Lncenc1 down-modulation across the different experimental conditions revealed a similar, significant impact on the expression of about 1000 genes (LRT and FDR < 0.05) (Figure 3C). Of note, higher variations were elicited by the shRNAs as compared to the ASO, as expected from the relative silencing strength and supporting results specificity. In agreement with the observed phenotype, upregulated genes were mostly related to embryonic development and cell fate commitment (Figure 3D and Supplementary Table 2), including the mesoderm and neuroectoderm markers Sox1, Nestin, Fgf5 and Foxd3 (Supplementary Table 2). In contrast, many down-regulated genes belonged to metabolic categories, including the glycolytic process (Figure 3D and Supplementary Table 2).

### Lncenc1 predominantly localizes to the cytosol and acts via miRNA-mediated mechanisms

**Figure 4:**
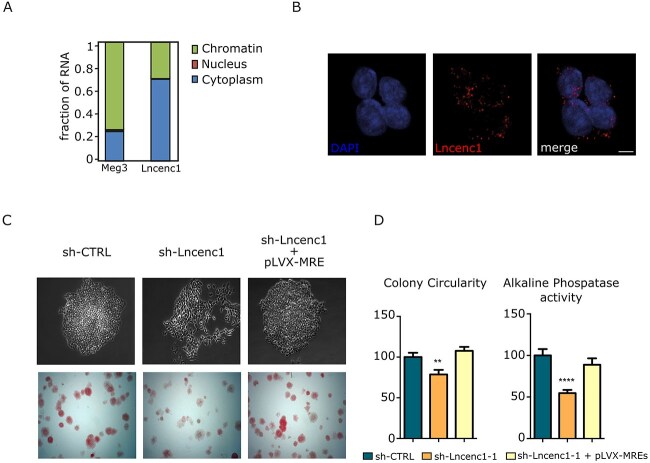
Lncenc1 localization and functional assessment. (**A**) Lncenc1 RNA was quantified by RT-qPCR on total RNA extracted from the indicated subcellular fractions of E14 cells, and the percentage of RNA localized to the indicated fractions is shown. The prevalently chromatinic Meg3 lincRNA and the cytosolic Actin mRNA (not shown) were also measured as controls. (**B**) Lncenc1 RNA-FISH and DAPI staining in E14 cells. (**C**) The differentiation triggered by Lncenc1 silencing in E14 cells was rescued by co-transfecting the pLVX-MRE construct, overexpressing the Lncenc1 MRE region, together with a Lncenc1 shRNA. Top panel: phase contrast imaging of representative colonies. Bottom panel: staining for alkaline phosphatase activity. (**D**) Quantification of morphology disruption (Colony Circularity) and of alkaline phosphatase staining. Data are shown as mean ± SEM of at least three independent experiments. ^*^^*^P < 0.001, ^****^P < 0.0001.

LincRNA functions are partly dictated by their cellular localization. To determine Lncenc1 localization, cytosolic, nuclear or chromatin E14 subcellular fractions were analyzed by RT-qPCR, showing prevalent cytosolic localization (Figure 4A). The predominantly chromatinic Meg3 lincRNA was used as a control. Confirming the fractionation data, FISH experiments showed a punctate, mostly cytosolic pattern (Figure 4B).

**Figure 5:**
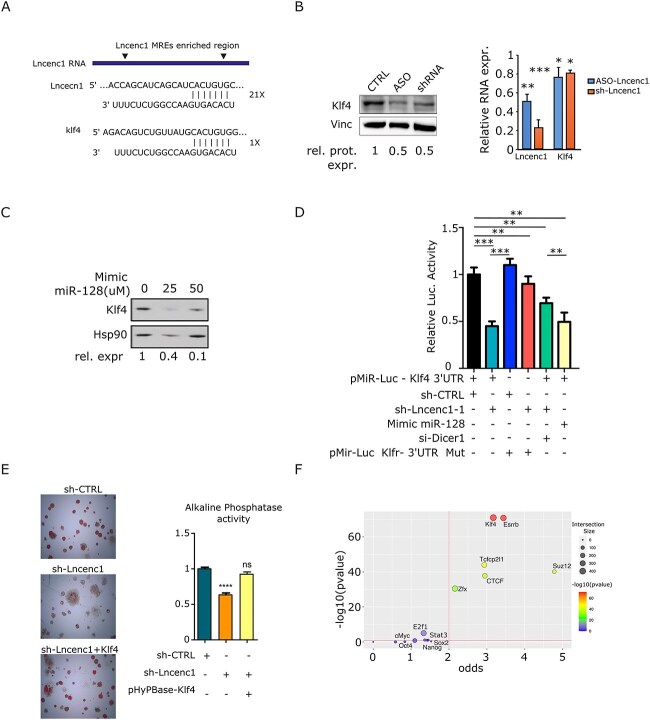
Lncenc1 regulates KLF4 expression by sequestering miR-128. (**A**) Schematic of Lncenc1 RNA, showing the MRE-enriched region between the arrowheads. Below, representative seed-pairing sites for miR-128 in the Lncenc1 RNA and the Klf4 3’UTR are shown. (**B**) Klf4 protein and RNA levels were measured in E14 ESCs, silenced or not for Lncenc1 by the indicated treatments. Numbers below the Western blot panel represent the relative Klf4 expression upon normalization to the Vinculin internal control, representative of three independent experiments.Ffold reduction was 50% +/− 5% (ASO) and 50% +/− 4% (shRNA). The histograms represent Lncenc1 and Klf4 RNA levels measured by RT-qPCR in the same samples. Data are mean ± SD of three independent replicates. Values were normalized to the 18S internal control and represented as fold change relative to control cells. The asterisks indicate statistically significant differences (two-tailed paired *t*-test) between the indicated groups and corresponding control; ^*^P < 0.01; ^*^^*^P < 0.001; ^*^^*^^*^P < 0.0001. (**C**) Western blot showing Klf4 protein downregulation upon miR-128 mimic transfection in E14 ESCs. Numbers below the panel represent the relative Klf4 expression upon normalization to the HSP90 internal control. Representative of three independent experiments. (**D**) Dual luciferase assay in E14 cells transiently co-transfected with the Renilla and the pMIRLuc-Klf4 3′UTR/Mut vectors, together with either control or Lncenc1 shRNA, the miR-128 mimic or Dicer1/control siRNA. Data are mean ± SD of at least three independent experiments. Asterisks indicate statistically significant differences between the indicated groups. ^*^^*^^*^P < 0.0001; ^*^^*^P < 0.001. (**E**) E14 cells stably expressing Klf4 and silenced or not for Lncenc1 were stained for Alkaline Phosphatase activity. The quantification shows mean ± SEM of at least three independent experiments. ^*^^*^^*^^*^P < 0.0001; ns, not significant. (**F**) Scatter plot showing the *in vivo* binding enrichment of transcription factors known to be active in mES cells (see Materials and Methods) at promoters of genes downregulated upon Lncenc1 silencing. Each dot represents a TF. The *Y*-axis shows the log transformed (−log10) significance of enrichment compared to random peaks computed using one-sided Fishers exact test. The *X*-axis represents the odds of finding a given number of promoters overlapping with a given TF. The size of the dots represents the intersection size. The indicated TFs were considered enriched if falling within the top right area delineated by dotted lines.

A potential mode of action of cytoplasmic lncRNAs is that of acting via miRNA-mediated mechanisms. Indeed, Lncenc1 exon 2 is highly enriched for canonical miRNA responsive elements (MREs). In particular, 22 and 17 elements were detected for microRNAs 128 and 138 MREs, respectively, with a highly significant enrichment (*P* < 1E^−150^, Supplementary Figure 3 and Supplementary Table 3). Of note, 21 out of the 22 miR-128 MREs are of the 7mer-8 type, containing a perfect match to the 6 nucleotides of the miRNA seed plus a further match at position 8. Conversely, 12 out of the 17 miR-138 MREs are of the 8mer-1a type, showing the seed match flanked by both a match at position 8 and an A at position 1, while 5 are 7mer-1a, with a seed match and an A at target position 1b [33]. Interestingly, 20 out of the 22 miR-128 MREs display a regular 22 nucleotides spacing, suggestive of a functional role. Both miRNAs are expressed in naive mESCs but also at different stages of differentiation toward the neuronal lineage, to become then enriched in the brain [34]. Together with its cytoplasmic localization, the Lncenc1 high MRE content suggests that its functions in maintaining ESCs naïve state could be at least partly due to buffering the activities of microRNAs, in particular miR-128 and miR-138, protecting their target mRNAs from silencing. Supporting this idea, we could show that the MRE-containing region is responsible for ESCs pluripotency maintenance, since its ectopic expression in E14 cells was sufficient to completely rescue the differentiation triggered by Lncenc1 silencing (Figure 4C, D and Supplementary Figure 4). Of note, miR138 levels were significantly affected upon Lncenc1 depletion, while miR-128 expression was not affected (Supplementary Figure 5). This is not surprising, since sponges are known to inhibit miRNAs activity by sequestering them from their endogenous targets, not necessarily implicating their degradation/downregulation.

### Lncenc1 acts by preventing miRNA-mediated suppression of Klf4 protein

In order to identify candidate Lncenc1 partner mRNAs, we took advantage of the prediction algorithm described in Karreth *et al.* (2015) [35], which is based on the relative expression of the different MRE-containing RNAs. For each mRNA sharing at least one MRE with Lncenc1, a score was computed that indicated the probability of being susceptible to Lncenc1 expression fluctuations. Only mRNAs and microRNAs actually expressed in mESCs were considered, based on available small RNA-seq data [36]. The transcript pairs were then ranked by decreasing score (Supplementary Table 4). Interestingly, several putative mRNA partners are well known pluripotency genes, with the core pluripotency factor Klf4 sharing four of the Lncenc1 MREs including one for miR128 (Figure 5A and Supplementary Table 4). Accordingly, Lncenc1 silencing could significantly downregulate Klf4 protein levels, with a lower reduction at the mRNA level (Figure 5B). These data support the idea of Lncenc1 acting in the cytoplasm via microRNA-mediated mechanisms, contributing to maintain high Klf4 expression. Confirming the functional role of miR128 in Klf4 regulation, transfection with a miR-128 mimic could significantly downregulate Klf4 protein levels in E14 cells (Figure 5C). In order to assess whether Klf4 mRNA is a direct target of miR-128, the activity of which is in turn inhibited by Lncenc1, we generated luciferase reporter vectors carrying the Klf4 3’UTR region, either wild type or mutated in the miR-128 MRE (Supplementary Figure 6), and assessed luciferase activity upon transient transfection in E14 ESCs (Figure 5D). Lncenc1 silencing resulted in a significant reduction of luciferase expression levels with respect to the non-targeting control plasmid, and this inhibitory effect was completely lost when the miR-128 MRE was disrupted, supporting the idea of Klf4 mRNA being a direct target for this microRNA, the availability of which is, in turn, regulated by Lncenc1 levels. Further confirming an RNA interference-based mechanism, Lncenc1-mediated modulation of luciferase activity was partially abolished by Dicer1 silencing (Figure 5D and Supplementary Figure 7A). Accordingly, the expression of an miR-128 mimic could recapitulate the effects of Lncenc1 silencing on luciferase expression (Figure 5D). Finally, overexpression of the MRE region could also rescue the downregulation of Klf4 protein levels triggered by Lncenc1 silencing (Supplementary Figure 7B). Taken together, these data demonstrate that Lncenc1 contributes to the maintenance of ESCs identity at least partly by protecting Klf4 mRNA from miR-128-mediated silencing. Although our results do not exclude the implication of other target mRNAs in addition to Klf4 in the Lncenc1-microRNA-mRNA network, downregulation of this key pluripotency transcription factor may well explain the differentiation phenotype triggered by Lncenc1 silencing. In perfect agreement, the phenotypic effects of Lncenc1 silencing were completely rescued by Klf4 ectopical expression (Figure 5E). Moreover, among all transcription factors known to be active in ES cells, Klf4 is the one able to bind the highest number of genes down-regulated upon Lncenc1 interference, as shown by ChIP-Seq analysis (Figure 5F), supporting a central role for Klf4 downregulation in determining the gene expression patterns following Lincenc1 silencing.

## DISCUSSION

Pluripotent ESCs represent valid systems to model mechanisms controlling stemness *versus* differentiation, with important implications in development, regenerative biology and cancer. To fully exploit their potential, however, the complete understanding of the molecular mechanisms underlying stemness is crucial. ESCs pluripotency can be maintained thanks to an intricated network of signaling pathways and transcription factors. Among these, the LIF–STAT3 pathway plays a crucial role in both human and mouse ESCs to elicit a ground state of pluripotency akin to the early epiblast state of the pre-implantation embryos [13, 15].

Our bioinformatic approach has allowed us to identify a set of STAT3-dependent lincRNAs abundantly and specifically expressed in mESCs (LincS). One of them, LincS3/Lncenc1, had already been described as involved in maintaining ESCs pluripotency [28, 30]. Our data indicate that Lncenc1 is indeed a direct STAT3 transcriptional target and has a functional impact on the maintenance of the pluripotent state of ESCs, as shown by cell differentiation upon Lncenc1 silencing. Notably, in addition to being required for LIF-dependent Lncenc1 expression, STAT3 is also able to drive Lncenc1 transcription independently of LIF.

We could show that Lncenc1 localizes predominantly to the cytoplasm by both cell fractionation and *in situ* hybridization experiments. Nevertheless, a small fraction could also be observed in the nuclear/chromatin fraction, suggesting that this ncRNA might act both in the nucleus and in the cytoplasm. Accordingly, Sun and colleagues have suggested that Lncenc1 may regulate the transcription of several genes of the glycolytic pathway [30]. Our own transcriptomic data also revealed perturbation of the glycolysis pathway upon prolonged Lncenc1 silencing but only at the late time point, suggesting an indirect mechanism. At any rate, as the ability of Lncenc1 transcriptional regulator STAT3 to regulate glucose metabolism is well-known [37, 38], an involvement of Lncenc1 in glycolysis regulation is not surprising. In turn, glycolysis plays an important role in fueling the high levels of histones acetylation required to ensure mouse stem cell plasticity [39]. Thus, the reduced glycolysis levels triggered by Lncenc1 silencing are likely to contribute to the observed loss of pluripotency.

The cytosolic localization of Lncenc1 as well as its high content in canonical MREs suggested miRNA-mediated mechanism(s). This hypothesis was confirmed by the observation that the expression of the MRE-containing region could fully rescue mESCs differentiation elicited by Lncenc1 silencing. Our experiments demonstrated that miR-128, 22 MREs of which are contained in the Lncenc1 largest exon, is an important regulator of the expression of the pluripotency transcription factor Klf4, which is indeed significantly down-regulated by Lncenc1 silencing. Indeed, sensitivity to Lncenc1 silencing was conferred to a luciferase reporter gene by the wild type Klf4 3’UTR, but not by a 3’UTR where miR-128 MRE was mutated. We therefore concluded that Lncenc1 functions as an endogenous miR-128 sponge, with Klf4 as one of its main targets. Therefore, Lncenc1 represents a positive feedback regulator of the LIF–STAT3 axis, since it is induced by STAT3 and contributes to maintain high expression levels of its direct transcriptional target Klf4. Our finding that Klf4 ectopic expression could rescue the differentiation phenotype induced by Lncenc1 silencing further strengthens this idea.

The expression of miR-138, the second microRNA which MREs are enriched in Lncenc1 RNA, is relatively low in mESCs and significantly induced during their differentiation toward the neuronal lineage [34], suggesting that Lncenc1 high expression levels may contribute to tame neural differentiation in pluripotent mESCs, unleashed then by simultaneous Lncenc1 down-regulation and miR-138 induction. Accordingly, transcriptome profiling of Lncenc1-silenced ESCs showed induced expression of ectoderm markers.

Our analysis revealed a number of potential Lncenc1 mRNA/miRNA partners, which may, at different degrees, contribute to Lncenc1-mediated regulation of the equilibrium between ESCs pluripotency and differentiation states. It is tempting to speculate that Lncenc1 and its miRNA partners may be involved in fine tuning the fluctuations between ESCs naive and primed states. Accurate quantification of their relative expression together with that of the involved microRNAs in single cell sequencing experiments will be instrumental to gain a deeper insight into this issue. Interestingly, predicted mRNA targets for miR128, but not for miR-138, were significantly enriched (Fisher exact test, *P* = 1.1E^−10^) among the genes significantly down-regulated upon Lncenc1 silencing.

Our observations are in agreement with the knowledge that the progressive effects of microRNAs on their target genes are crucial players in the process of gene expression canalization typical of early development [40], a phenomenon in which also the regulation of their activity by sponging RNAs should be factored in. Our data may also help further dissecting the well-known pro-oncogenic roles of STAT3, suggesting that aberrant, ectopic activation of Lncenc1 and perhaps other pluripotency-associated non-coding STAT3 target RNAs may contribute to the acquisition of cancer stem cell features.

**Key Points**

- Identification of ES cells-specific/STAT3-dependent long non-coding RNAs potentially mediating STAT3-induced pluripotency.
- LIF–STAT3 signaling controls naive pluripotency via a lincRNA–microRNA circuit.
- Post-transcriptional control of Klf4 expression via STAT3-regulated microRNA activity.
- The STAT3-dependent ES cells-specific Linenc1 RNA controls Klf4 expression and pluripotency acting as a sponge toward microRNAs targeting Klf4.

## Supplementary Material

Supplementary_Table_I_elad045

Supplementary_Table_II_elad045

Supplementary_Table_III_elad045

Supplementary_table_IV_elad045

Supplementary_table_V_elad045

Suppl_Figs+legends_VP_17-8_elad045

## Notes

**Emanuele Monteleone** is an assistant professor in bioinformatics at the University Vita-Salute San Raffaele in Milan, Italy. He received his Ph.D. in Molecular Medicine at the University of Torino. Dr. Monteleone’s current research focuses in the organization and function of chromatin in cell identity transitions in both physiological and pathological conditions. In particular, he is interested in nucleosome dynamics and positioning in key cellular processes, including reprogramming to iPS cells, Epithelial-To-Mesenchymal Transition and cellular senescence.

**Paola Corrieri** after a Master Degree in Preclinical and Clinical Research and Drug development, she won a fellowship and worked as study coordinator. She is currently a Clinical Research Associate, supporting sites into clinical trials development. She is working on studies in the following fields: oncology, infectious diseases and hemoglobinopaties.

**Paolo Provero** is a professor of Molecular Biology in the Department of Neurosciences of the University of Turin. He received his PhD degree in Theoretical Physics from the University of Genoa in 1992. Since 2000 his research has focused on computational genomics, and in particular on the development of analytical methods for the study of gene regulation at the transcriptional and post-transcriptional levels.

**Daniele Viavattene** is a PhD student in Molecular Medicine at the Molecular Biotechnology Center, University of Turin, Italy. He received his M.S.degree in Medical Biotechnology at University of Turin and his B.S. degree in Biotechnology at University of Messina, Italy. His research interests lie in cancer and molecularbiology.

**Lorenzo Pulvirenti** is a clinical data manager at AOU S. Luigi Gonzaga at the department of oncology. He received his M.D. in molecular biology at the University of Turin. His research intersects lie in clinical data management in the field of oncology.

**Laura Raggi** is a third year PhD student at San Raffaele Telethon Institute for gene therapy (SR-TIGET) in Milan, Italy. Her research interests focus on hematopoietic stem cell-niche interactions in beta-thalassemia with potential translational relevance for improving gene therapy in congenital anemia.

**Elena Carbognin** is an Assistant Professor at the Department of Biology, University of Padua, Italy. She received her Ph.D. degree in Biosciences at Padua University, Italy. Her research focuses on the molecular mechanisms controlling maintenance of pluripotency in embryonic stem cells and early differentiation.

**Marco E. Bianchi** is Professor of Molecular Biology at Vita-Salute San Raffaele University in Milano, Italy. He is most interested in inflammation, both in trauma and cancer, and in epigenetics. He first found that High Mobility Group Box 1 protein (HMGB1) is a DNA chaperone that both recognizes and induces DNA bending. He then realized that nuclear HMGB1 can be passively released or actively secreted by dead or stressed cells, and serves as a Damage Associated Molecular Pattern (DAMP). DAMPs are signals of tissue distress that Polly Matzinger had hypothesized in her “Danger Theory” to initiate immune responses but had not been identified experimentally. He next showed that HMGB1 has multiple redox forms that bind different receptors. The switching of HMGB1 between its redox forms is critical for the damaged tissue to decide to continue inflammation or start healing. In cancer as well, HMGB1 redox forms support tumor growth or instruct immune responses, for example in Immunogenic Cell Death. He also demonstrated that the number of nucleosomes is actively controlled by the cells.

**Graziano Martello** is Associate Professor at the Department of Biology, University of Padua. He received in 2009 a Ph.D. in Genetics and Developmental Biology studying the role of non-coding RNAs during early development. He then moved to Cambridge University (UK) to study pluripotent stem cells from 2010 to 2014. He set up his independent laboratory of pluripotent stem cell biology (martellolab.org) at University of Padua in 2014, where he was Associated Professor of Molecular Biology in 2017. His research is focussed on understanding the mechanisms controlling pluripotent maintenance and induction, and to model neurodegenerative diseases with pluripotent cells. His work is supported by the Armenise-Harvard foundation, Telethon foundation, Microsoft Research and the European Research Council.

**Salvatore Oliviero** is a Professor in Molecular Biology at the University of Torino and head of the Laboratory of Epigenetics at the Italian Institute for Genomic Medicine (IIGM). Between 1985 and 1988 he was at EMBL (Heidelberg, Germany) as a PhD student. From 1988 to 1992 he was postdoctoral fellow at Harvard Medical School (Boston, USA). His laboratory identified and characterized a new vascular endothelial angiogenic growth factor (VEGF-D); unveiled the molecular mechanism by which MYC induces the elongation step of the transcription. More recently his laboratory is focused on the function of epigenetic modifications that regulate the early steps of embryonic development.

**Pier Paolo Pandolfi** is an Italian scientist renowned for his groundbreaking contributions to the field of cancer research and molecular medicine. Pandolfi completed his medical degree at the University of Perugia in Italy and subsequently pursued a Ph.D. in Molecular and Cellular Biology at the European Molecular Biology Laboratory (EMBL) in Germany. He became PI at the to the Memorial Sloan-Kettering Cancer Center, NY, USA in 1994, and then joined the Harvard Medical School to become Director of the BIDMC Cancer Center in 2013. His research focuses on the genetic factors underlying human cancers.

**Valeria Poli** is Professor in Molecular Biology at the University of Torino, Italy since 2001. She was Post-doctoral fellow at the EMBL, Heidelberg, Germany in 1988-1990 and at Columbia University, NY, USA in 1990-1992. She was Principal Investigator at IRBM, Pomezia, Rome, Italy in 1992-1997, and at the University of Dundee, Scotland, UK in 1997-2001. She has cloned the IL-6-acivated transcription factor C/EBPbeta and generated and characterized several genetically modified mouse strains including IL-6 and C/EBPbeta null, and conditional and isoform-specific STAT3 null. Her research focuses on the regulation of gene expression during inflammation, auto-immunity and cancer.
