# Correction to: STAT3-dependent long non-coding RNA Lncenc1 contributes to mouse ES cells pluripotency via stabilizing Klf4 mRNA

**DOI:** 10.1093/bfgp/elad047

**Published:** 2023-10-18

This is a correction to: Emanuele Monteleone, Paola Corrieri, Paolo Provero, Daniele Viavattene, Lorenzo Pulvirenti, Laura Raggi, Elena Carbognin, Marco E Bianchi, Graziano Martello, Salvatore Oliviero, Pier Paolo Pandolfi, Valeria Poli, STAT3-dependent long non-coding RNA Lncenc1 contributes to mouse ES cells pluripotency via stabilizing Klf4 mRNA, *Briefings in Functional Genomics*, 2023; elad045, https://doi.org/10.1093/bfgp/elad045

In the originally published version of this manuscript, there was a typographical error within the title. This should read: ``STAT3-dependent long non-coding RNA Lncenc1 contributes to mouse ES cells pluripotency via stabilizing Klf4 mRNA'' instead of: ``STAT3-dependent long non-coding RNA Lncenc1 contributes to mouse ES cells pluripotency via stabilizing K mRNA''.

This error has been corrected in the article.

