# Correction to: STAT3-dependent long non-coding RNA Lncenc1 contributes to mouse ES cells pluripotency via stabilizing Klf4 mRNA

**DOI:** 10.1093/bfgp/elaf012

**Published:** 2025-07-15

This is a correction to: Emanuele Monteleone, Paola Corrieri, Paolo Provero, Daniele Viavattene, Lorenzo Pulvirenti, Laura Raggi, Elena Carbognin, Marco E Bianchi, Graziano Martello, Salvatore Oliviero, Pier Paolo Pandolfi, Valeria Poli, STAT3-dependent long non-coding RNA Lncenc1 contributes to mouse ES cells pluripotency via stabilizing Klf4 mRNA, *Briefings in Functional Genomics*, Volume 23, Issue 5, September 2024, Pages 651–662, https://doi.org/10.1093/bfgp/elad045

The following changes have been made to the originally-published author details. The affiliations for the first author Emanuele Monteleone are emended to read:

"Department of Molecular Biotechnology and Health Science, University of Torino, Via Nizza 52, 10126 Torino, Italy

Università Vita-Salute San Raffaele and l'IRCCS San Raffaele Scientific Institute, Milan, Italy''.

Those for the eighth author, Marco Bianchi, are emended to read:

``Università Vita-Salute San Raffaele and l'IRCCS San Raffaele Scientific Institute, Milan, Italy''.

In both the PDF and online publications of the paper, the author biography has been added for Dr Marco Bianchi.

