# Supplementary material for: STAT3-dependent long non-coding RNA Lncenc1 contributes to mouse ES cells pluripotency via stabilizing Klf4 mRNA
Source: Brief Funct Genomics. 2023 Oct 4;23(5):651–62. doi: 10.1093/bfgp/elad045 (PMC11428181; doi:10.1093/bfgp/elad045)
Supplement: Suppl_Figs+legends_VP_17-8_elad045 [file suppl_figs+legends_vp_17-8_elad045.docx]

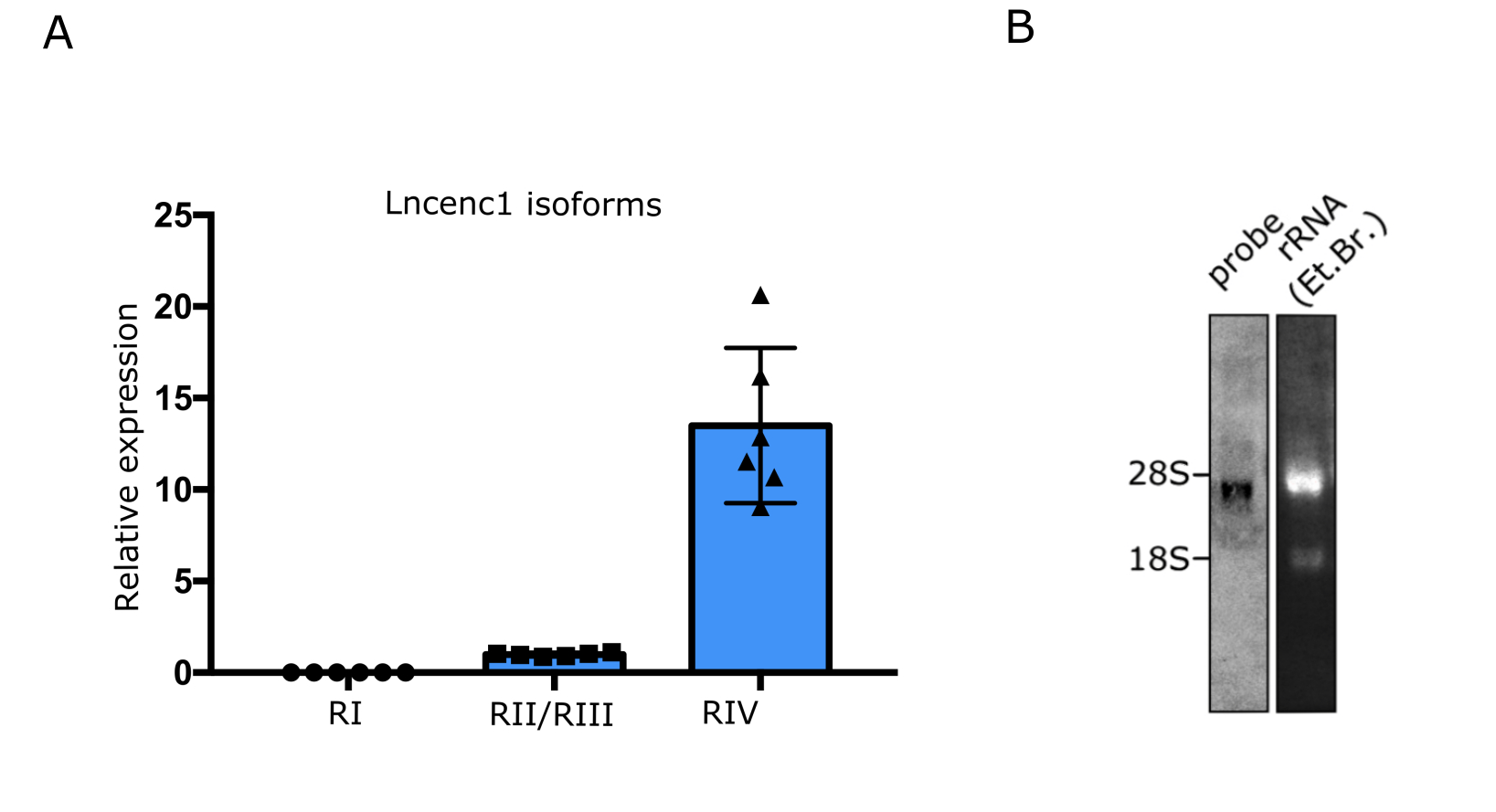


**Supplementary Figure 1**

(A) Relative expression of Lincenc1 isoforms. Measures were evaluated using specific primers targeting the three RefSeq curated isoforms (RI,RII and RIII) and a novel and prevalent isoform (RIV). Note that the primers amplifying RIV can also amplify RIII, which is however expressed at very low levels as shown by the product of RII/RIII primers. qRT-PCR data are represented as the mean of replicates ± SD of values normalized to the TBP internal control.

(B) Lncenc1 RNA was analyzed by Northern blot on E14 total RNA. The Ethidium Bromide staining of the nylon membrane (right lane) shows mature 28S and 18S rRNAs as molecular weight markers.


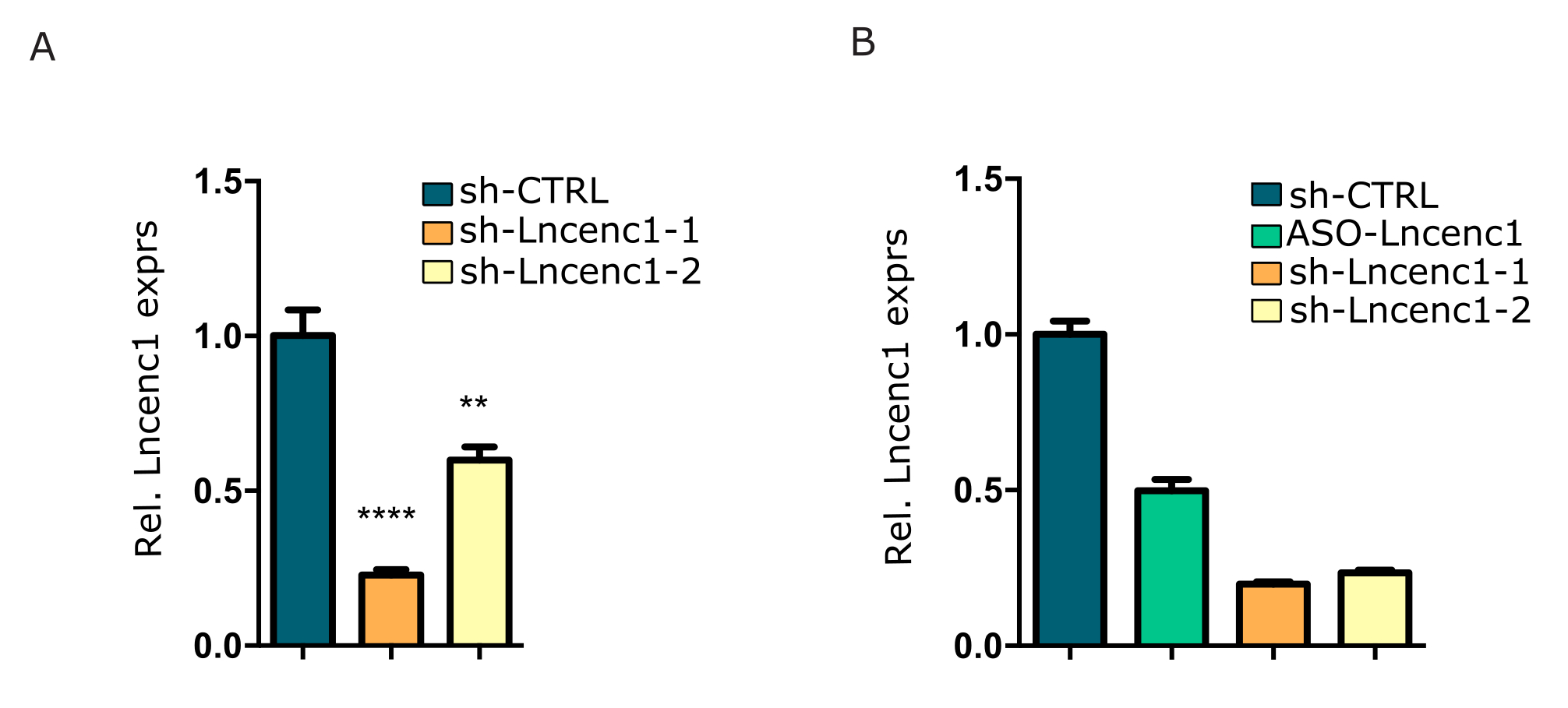


**Supplementary Figure 2**

E14 cells were transfected with either control or Lncenc1 targeting shRNAs (72 hours) and/or ASO (36 hours), as indicated (sh-CTRL, sh-Lncenc1-1, sh-Lncenc1-2, ASO). (A) refers to the experiment of Fig. 2A, and B to the sequencing of Fig. 2B. Silencing was evaluated by qRT-PCR upon normalization to TBP mRNA as an internal control. Statistically significant differences with respect to the control condition (A, n=3) are shown. ****, p<0.0001, **, p<0.01.


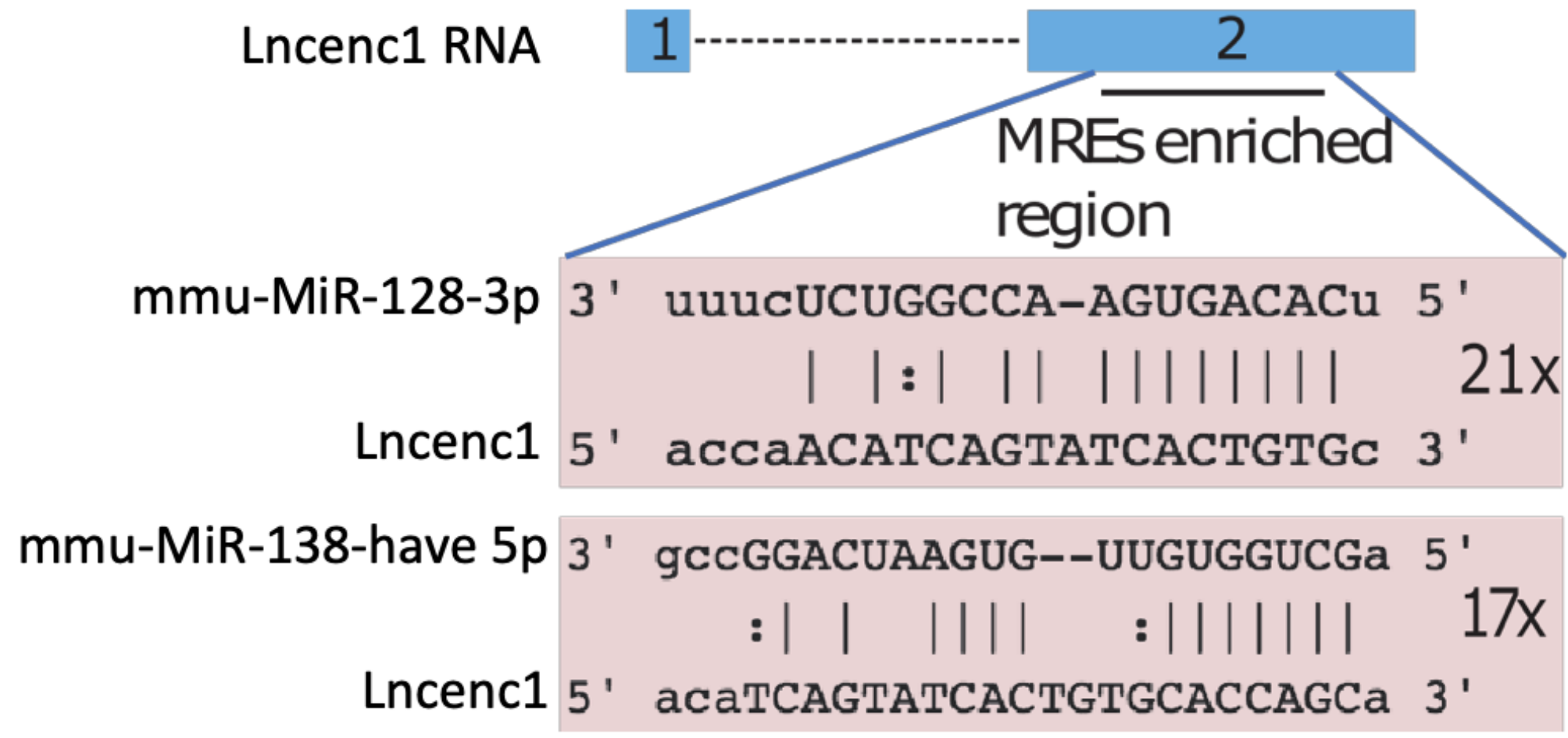


**Supplementary Figure 3**

Schematic of the Linenc1 locus showing the MRE-enriched region. Representative

seed-pairing sites for miR-128 and miR138 are shown.


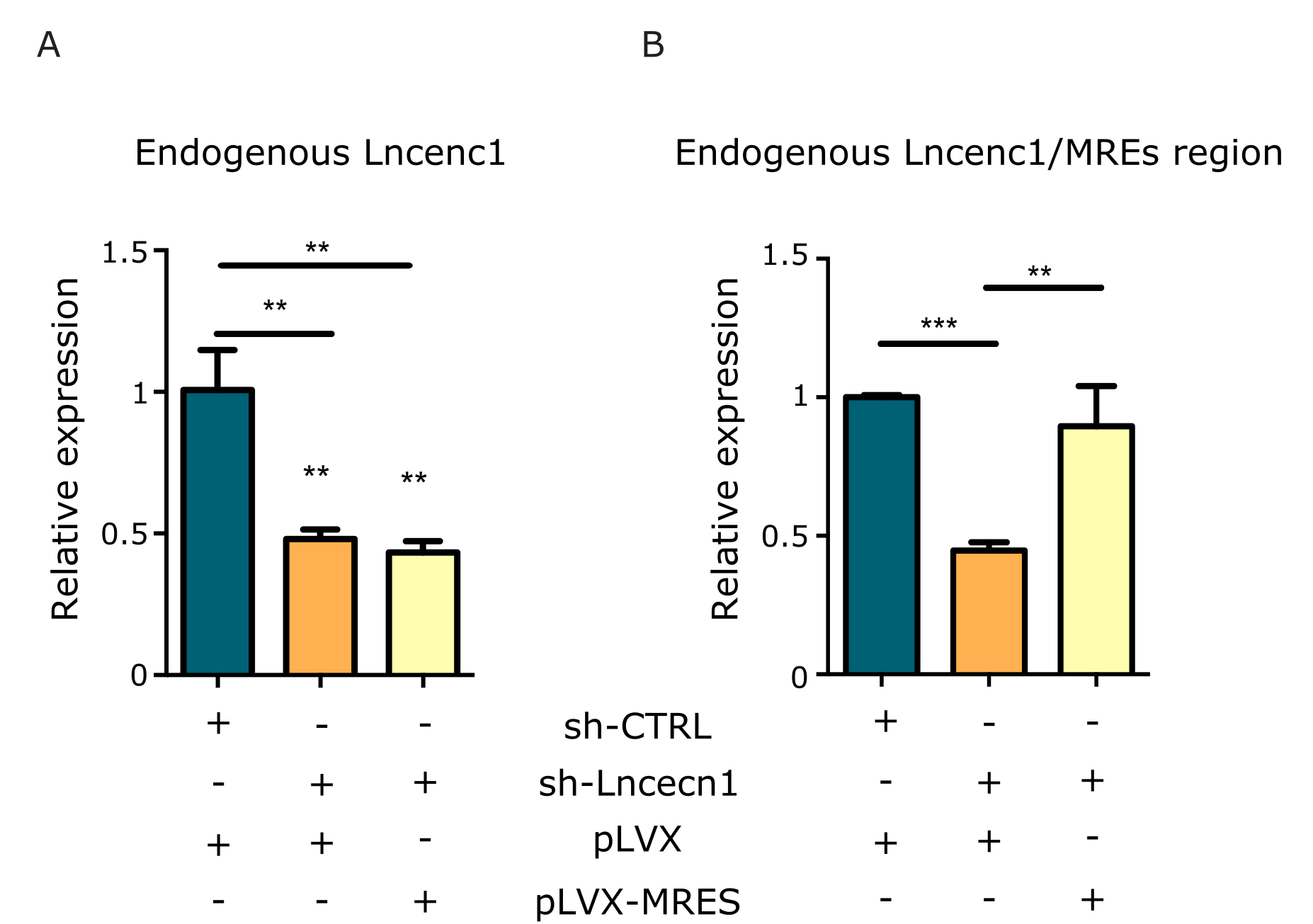


**Supplementary Figure 4**

E14 ESCs were co-transfected with shRNA-expressing plasmids (sh-CTRL, sh-Lncenc1-1) and a PLVX vector either empty or expressing the Lncenc1 MREs region (PLVX-MREs). The expression of either the endogenous Lncenc1 (**A**) or the MRE region only (**B**) was evaluated by qRT-PCR with specific sets of primers (Materials and Methods), upon normalization to the TBP mRNA as an internal control [mean +/- SD]. Statistically significant differences with respect to the control condition are shown. ***, p<0.001, **, p<0.01.


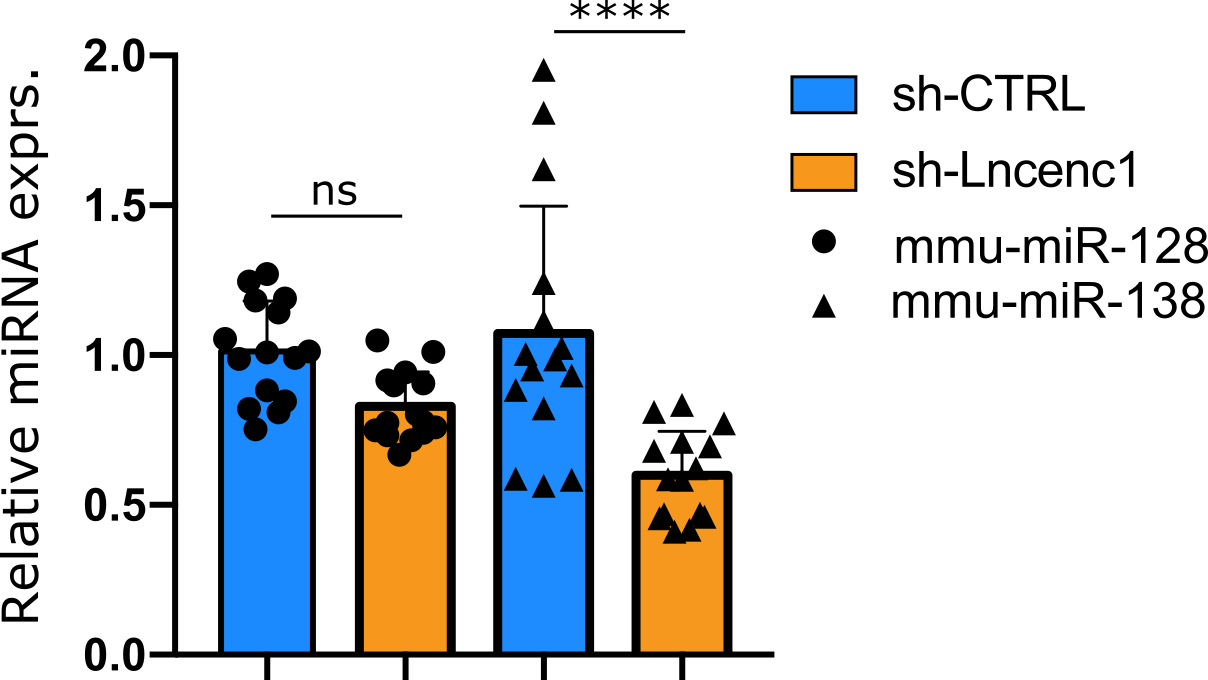


**Supplementary Figure 5**

E14 cells were transfected with either control or Lncenc1 targeting shRNAs (72 hours). The expression of either miR-128 or miRNA-138 was evaluated by qRT-PCR with specific sets of primers (Materials and Methods), upon normalization to the U6 RNA as an internal control [mean +/- SD]. Statistically significant differences with respect to the control condition are shown. ****, p<0.0001.

**
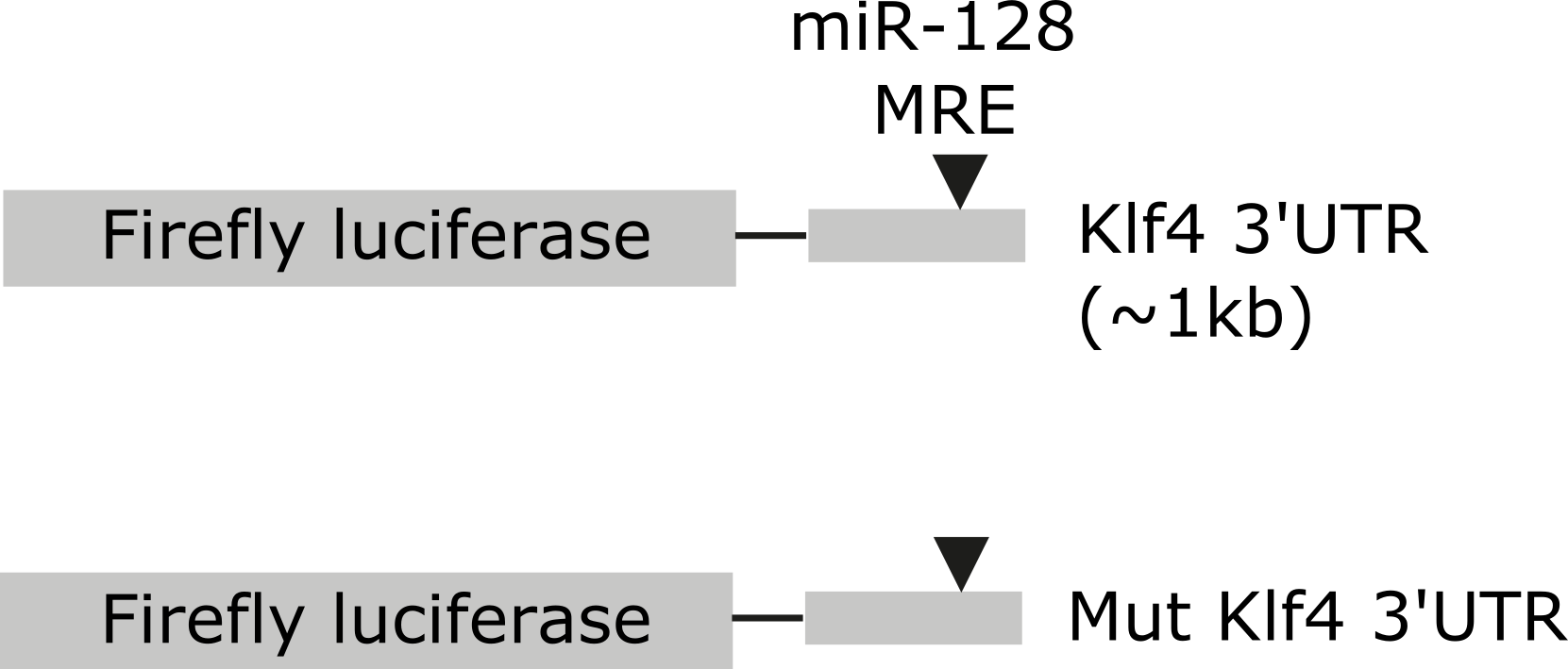
**

**Supplementary Figure 6**

Schematic representation of the luciferase constructs carrying the Klf4 3′UTR, either wild type or mutated in the miR-128 MRE (Mut-Klf4 3’UTR).


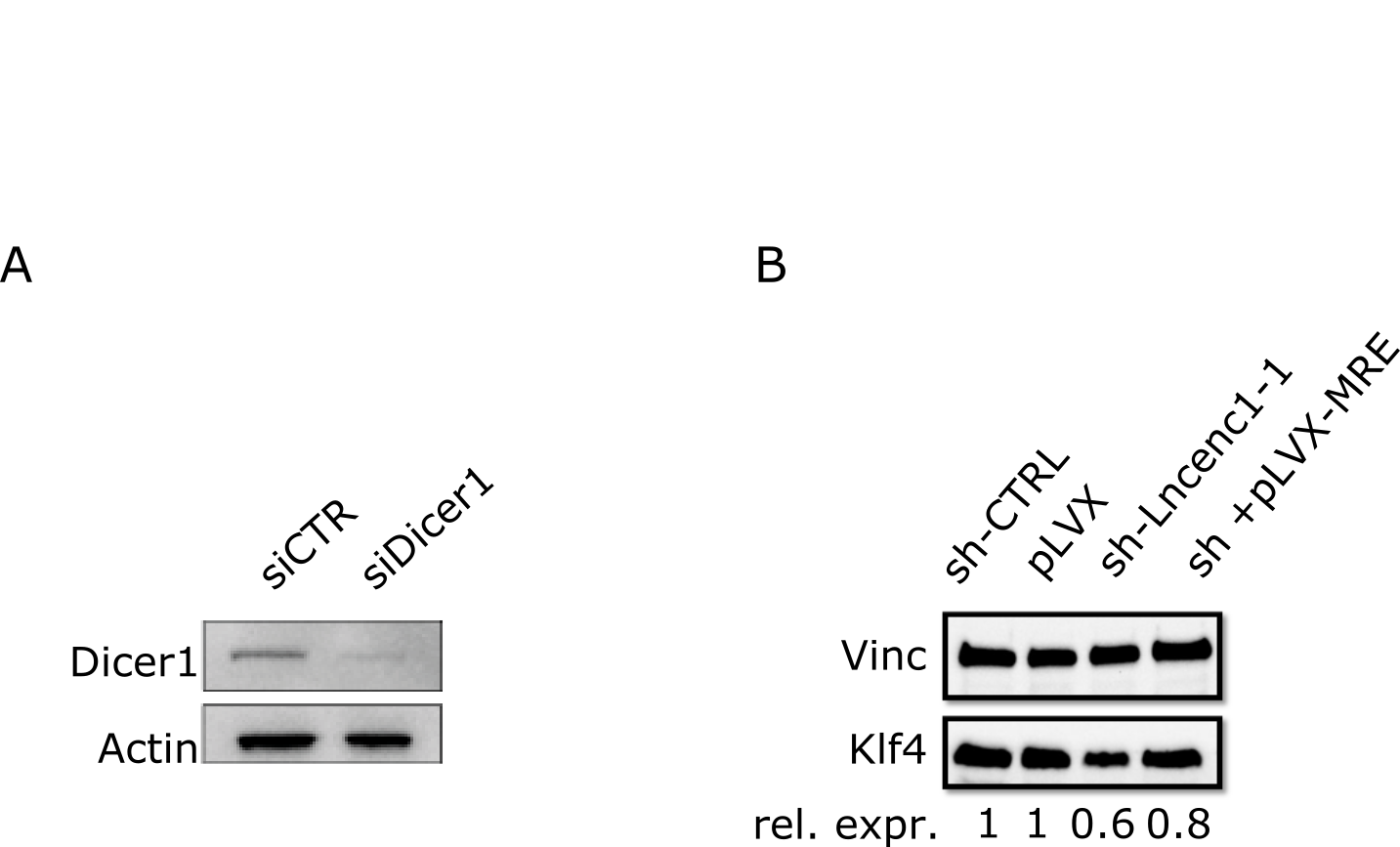


**Supplementary Figure 7**

(**A**) Western blot analysis showing knockdown efficiency of Dicer1 upon siRNA transfection in E14 cells. (**B**) Western blot showing partial rescue of the Klf4 downregulation upon Lncenc1 silencing (sh-Lncenc1 vs sh-CTRL) by overexpression of the Lincenc1 MRE region (pLVX-MRE) in E14 cells. The numbers represent the relative Klf4 expression upon normalization to the Vinculin internal control. Representative of three independent experiments.
